# Enhancing Bioaerosol Sampling by Andersen Impactors Using Mineral-Oil-Spread Agar Plate

**DOI:** 10.1371/journal.pone.0056896

**Published:** 2013-02-27

**Authors:** Zhenqiang Xu, Kai Wei, Yan Wu, Fangxia Shen, Qi Chen, Mingzhen Li, Maosheng Yao

**Affiliations:** State Key Joint Laboratory of Environmental Simulation and Pollution Control, College of Environmental Sciences and Engineering, Peking University, Beijing, China; Dowling College, United States of America

## Abstract

As a bioaerosol sampling standard, Andersen type impactor is widely used since its invention in 1950s, including the investigation of the anthrax attacks in the United States in 2001. However, its related problems such as impaction and desiccation stress as well as particle bounce have not been solved. Here, we improved its biological collection efficiencies by plating a mineral oil layer (100 µL) onto the agar plate. An Andersen six-stage sampler and a BioStage impactor were tested with mineral-oil-spread agar plates in collecting indoor and outdoor bacterial and fungal aerosols. The effects of sampling times (5, 10 and 20 min) were also studied using the BioStage impactor when sampling environmental bioaerosols as well as aerosolized *Bacillus subtilis* (G+) and *Escherichia coli* (G-). In addition, particle bounce reduction by mineral-oil-plate was also investigated using an optical particle counter (OPC). Experimental results revealed that use of mineral-oil-spread agar plate can substantially enhance culturable bioaerosol recoveries by Andersen type impactors (p-values<0.05). The recovery enhancement was shown to depend on bioaerosol size, type, sampling time and environment. In general, more enhancements (extra 20%) were observed for last stage of the Andersen six-stage samplers compared to the BioStage impactor for 10 min sampling. When sampling aerosolized *B. subtilis*, *E. coli* and environmental aerosols, the enhancement was shown to increase with increasing sampling time, ranging from 50% increase at 5 min to ∼100% at 20 min. OPC results indicated that use of mineral oil can effectively reduce the particle bounce with an average of 66% for 10 min sampling. Our work suggests that enhancements for fungal aerosols were primarily attributed to the reduced impaction stress, while for bacterial aerosols reduced impaction, desiccation and particle bounce played major roles. The developed technology can readily enhance the agar-based techniques including those high volume portable samplers for bioaerosol monitoring.

## Introduction

Exposure to bioaerosols naturally occurring or intentionally released has been shown to cause various adverse health effects[1]–[6]. Apart from this, the roles of bioaerosol in atmospheric chemistry are also getting increased attention[7], [8]. For bioaerosol rated research, an important part is its sampling[9]. Following the discovery of airborne mold spores dated back to 1833, an impinger-like bioaerosol sampler was first invented and the relevant findings were published in the journal of *Science* [10]. Afterwards, a number of biological aerosol sampling devices were invented including an all-glass impinger [11], a device utilizing the electrical force[12], and a critical-orifice liquid Impinger [13]. In 1958, the six-stage Andersen sampler was invented[14], and further recommended as a standard bioaerosol sampling tool [15]. Due to its simplicity, high collection efficiency, and ease of use, Andersen-type impactor is still widely used in bioaerosol sampling, including the investigation of anthrax attacks in the United States in 2001[6].

Andersen-type impactor is inertia based and employed primarily in conjunction with an agar plate for sampling culturable bioaerosols. For impaction based sampler, its biological efficiency was shown to be a function of sampler cutoff size, jet-to-plate distance and microorganism[16], [17]. In addition to its overloading problems in sampling bioaerosols of high levels, its disadvantages including impaction stress, particle bounce, desiccation effects, and microbial embedding were also reported[18], [19]. The Andersen six-stage sampler has six stages, each of which has a different cutoff size, thus corresponding to different impaction velocities: 24, 12.8, 5.3, 3.0, 1.8, and 1.1 m/s. Accordingly, for different stages impaction stress is different. Among other Andersen type impactors, BioStage impactor (SKC), the sixth stage of Andersen six-stage sampler, is also widely used, including its portable version, QuickTake 30 (SKC). Use of bare agar plate for collecting culturable bioaerosols would dry agar surface over a prolonged sampling time, thus leading to the particle bounce. On the other hand, due to the high particle impaction velocity, the particle could be also embedded directly into the agar medium, which could inhibit their growth and detection[18], [19]. Besides, air desiccation also plays an important role in the decreased efficiencies when sampling bioaerosols by impactors [20]. Due to these problems, the Andersen sampler often underestimates the biological aerosol load. Unfortunately, no relevant improvements have been made in Andersen type samplers for bioaerosol sampling since its invention.

Mineral oil (without promoting or inhibiting bacterial growth) has been used as a collection medium for BioSampler (SKC) for extended sampling time as a means to reduce the reaerosolization problem [21]. It was shown that the BioSampler can be operated with the mineral oil for 8 hr without reduced collection efficiency [21]. In our previous study, we have improved the bioaerosol sampling for a portable bioaerosol sampler, RCS High Flow (Biotest), using mineral oil strip in replace of the agar strip where mineral oil is fully filled into those small cubes of the sampling strip [22]. Experimental results revealed that when sampling in different environments, use of mineral -oil-strip yielded significantly higher, about 4–12 times, culturable bacterial aerosol concentration levels compared to the use of agar strip [21]. Use of mineral-oil-strip results in less particle bounce due to its high viscosity and non-evaporating attributes. Besides, use of mineral oil could also reduce the desiccation on those bioaerosols already collected and embedded into the mineral oil. Nonetheless, the approach used in our previous work has its own limitation, i.e., after the sampling the mineral oil has to be taken out and plated on agar plate for bioaerosol detection. This extra step would lead to a loss of mineral oil on the strip in addition to possible contamination.

Here, we aimed to investigate the feasibility of using a mineral-oil-spread agar plate (plating a thin layer of mineral oil on agar plate), which avoids the extra sample process step, to improve the bioaerosol sampling by widely used Andersen type samplers. To investigate effects of different impaction velocities, an Andersen six-stage sampler was used. In addition, the effects of sampling time were also studied using a BioStage impactor, the sixth stage of Andersen sampler. In this work, both aerosolized sensitive bacteria *E. coli*, hardy species *B. subtilis* and environmental bacterial and fungal bioaerosols were collected using mineral-oil-spread agar plates and compared with those regular ones. The experimental results demonstrated that use of mineral-oil-spread agar plate can substantially enhance the biological collection efficiencies of the Andersen type samplers, and the developed technology can be immediately used in enhancing current agar plate based tools for bioaerosol monitoring.

## Materials and Methods

### Bioaerosol samplers used and preparation of mineral-oil-spread agar plate

In this study, an Andersen six-stage sampler (SKC) and a portable BioStage impactor (QuickTake 30) (SKC Inc., Eighty Four, PA) were used for studying the efficiencies of mineral-oil-spread agar plates. The Andersen six-stage sampler has six stages, which have respective cutoff sizes of 0.65, 1.1, 2.1, 3.3, 4.7 and 7.0 µm, and different impaction velocities of 24-1.1-m/s. The BioStage impactor is the sixth stage of the Andersen six-stage sampler, and has a cutoff size of 0.65 µm. Recent development of QuickTake 30 pump from SKC has equipped the BioStage impactor with the portability. The QuickTake 30 pump can continuously vacuum air for 4 h at the flow rate of up to 30 L/min (SKC Inc., Eighty Four, PA).

In this study, right before the sampling the mineral-oil-spread agar plates were prepared by first pipetting 100 µL mineral oil (ViaTrap Collection Medium, SKC) and then plating it evenly using a spreading bar onto Trypticase soy agar (TSA) (30 mL) (Becton Dickinson Microbiological System, Sparks, MD) Petri dish plate for bacteria, and Malt Extract Agar (30 mL) (Becton Dickinson Microbiology Systems, Sparks, MD) for fungi inside a Biosafety Level II cabinet (Sterilchem GARD, Baker Co., Sanford, Maine). Given the dimension (∼8.6 cm in diameter) of the Petri dish used, a mineral oil film of ∼180 µm thick was formed on the agar plate. The mineral oil used was indicated to neither kill nor grow bacteria [21].

### Experimental procedures

Here, the Andersen six-stage sampler and the BioStage impactor were operated separately at 28.3 L/min in conjunction with either mineral-oil-spread agar plates or regular ones for collecting bacterial and fungal aerosols in both indoor and outdoor environments. For use of the Andersen six-stage sampler, only 10 min sampling was tested; while for the BioStage impactor, different sampling times: 5, 10, and 20 min were studied. The use of mineral-oil-spread agar plates and regular ones were alternated, and at least three independent experiments were conducted in each of the environments studied. Such an alternation was performed three times over the tested sampling time periods for each set of experiments such that the described influences if any would be randomized and distributed between the mineral-oil-spread and regular agar plates. For the environmental sampling, we left the samplers alone when they were started for collecting bioaerosols, therefore there would be limited influence from people on the sampling.

To further investigate the technique, *Bacillus subtilis* (ATCC 9372) (vegetative cells) and *Escherichia coli* (ATCC 15597) were also aerosolized using a Collison nebulizer (BGI) at a nebulization flow rate of 2.5 L/min and the resulting bioaerosols were further carried by a nitrogen airflow (∼13 L/min) into a chamber placed inside the Biosafety Level II cabinet. *B. subtilis*, a hardy species, represents Gram-positive (G+) cells, while *E. coli*, a sensitive species, represents Gram-negative (G-) cells.The aerosolized bacterial species were then collected using the BioStage impactor at 28.3 L/min alternately with mineral-oil-spread agar plates and regular ones for 5, 10, and 20 min. The extra airflow for the BioStage impactor was compensated by the clean air inside the cabinet. After the sampling, all agar plates were cultured on Petri dishes with Trypticase Soy Agar (Becton, Dickson and Company, Sparks, MD) directly at 26 °C for environmental bioaerosols, at 37 °C for *E. coli* and at 30 °C for *B. subtilis*. Colony forming units (CFUs) were manually counted and statistically corrected. At least three independent experiments were conducted with each bacterial species. For the aerosolized bacterial species, they were in a small size range (0.6–1.0 µm) if not in aggregates, thus most of them would be collected by the last stage of the Andersen six-stage sampler. Accordingly, we only tested the BioStage impactor here under the controlled laboratory conditions. When performing the sampling, mineral-oil-spread and regular agar plates were brought to the sampling sites without the sampling as the blank controls.

In addition, an optical particle counter (OPC, GRIMM, model 1.108) was also used to monitor the particle bounce both for mineral-oil-spread agar plates and regular ones with the BioStage impactor. The OPC measures particle number concentration by optical size from 0.3 to 20 µm in 15 channels (GRIMM). The BioStage impactor was operated with a two-way tubing which connects to the OPC at 1.2 L/min and the vacuum pump at 28.3 L/min, respectively, over a 20-min time period. For the first 10 min sampling, mineral-oil-spread agar plate was used, while for next 10 min regular agar plate was used. The particle size distributions for each case were monitored at the downstream of the BioStage impactor by the OPC. The particle bounce reduction for each particle size range (0.35–0.45, 0.45–0.575, 0.575–0.725, 0.725–0.9, 0.9–1.3, 1.3–1.8, 1.8–2.5, 2.5–3.5, 3.5–4.5, 4.5–6.25, 6.25–8.75, 8.75–12.5, 12.5–17.5) was calculated using the following equation:

Particle bounce reduction = (1- *N_mineral-oil-spread_*/*N_regular_*)×100%

where *N_mineral-oil-spread_* is average particle concentration level for each specific size range when mineral-oil-spread agar plate is used, while *N_regular_* refers to the counterpart when the regular agar plate is used.

### Statistical analysis

The bioaerosol concentration levels obtained by mineral-oil-spread agar plates and those regular ones were checked for normality by Shapiro-Wilk test and further analyzed using independent sample t-tests through the SPSS 16.0 Software. The equality of variances of the variable was also checked using Levene's Test. A p-value of less than 0.05 indicates a significant difference between groups (confidence level 95%).

## Results and Discussion

**Figure 1:**
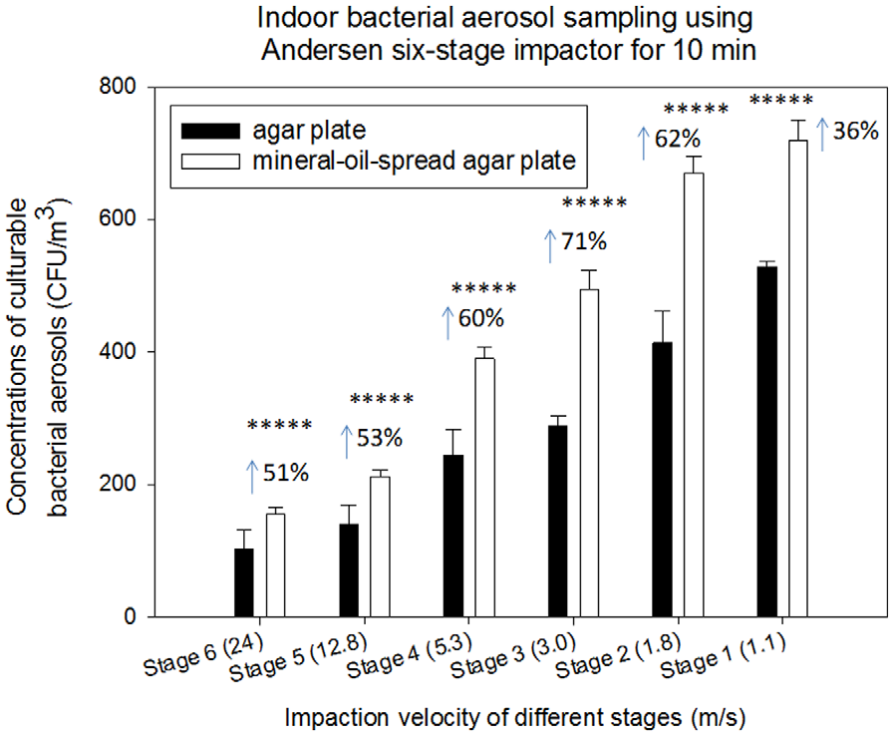
Size specific biological collection efficiencies of Andersen six-stage impactor together with agar plate and mineral-oil-spread agar plate in sampling bacterial aerosols at a sampling time of 10 min in an indoor environment; 100 µL mineral oil was evenly spread onto entire agar plate; 30 mL agar was used to fill the agar plate; data points represent averages and standard deviations of three independent sampling experiments; ***** indicates a statistically significant difference.

Here, we have investigated the feasibility of using mineral-oil-spread agar plate in enhancing the bioaerosol sampling by Andersen type samplers. In our experiments, we have observed that use of mineral-oil-spread agar plates substantially improved the biological collection efficiencies of Andersen type samplers for both environmental bioaerosols and aerosolized ones (p-values are shown in Table 1). Figure 1 shows the indoor bacterial aerosol sampling for 10 min using the Andersen six-stage sampler in conjunction with mineral-oil-spread and regular agar plates. As observed from the figure, regardless of the particle size ranges mineral-oil-spread agar plates were shown to substantially enhance the bacterial aerosol collection efficiencies of the Andersen six-stage sampler from 36% increase for the 6 th stage to 71% for the 3^rd^ stage (p-values<0.05 shown in Table 1). When sampling bacterial aerosols outdoors using the Andersen six-stage sampler, similar results were obtained as shown in Figure S1 (Supporting Information). Different from indoor bacterial aerosol sampling, the protective effect was found more pronounced for the stage 4 of the sampler, about 97% enhancement. This difference is likely due to the difference in particle size distribution and bacterial aerosol compositions in different environments. In general, the observed enhancements varied with stages of the Andersen six-stage sampler, which could be attributed to the differences in several factors: impaction stress, particle size, species composition and environments.

**Table 1:** Statistical analysis of performances of mineral-oil-spread agar together with Andersen six-stage sampler and BioStage impactor for sampling bioaerosols.

N = 4, p-values (two tailed) (independent sample t-test) When the BioStage impactor is used with mineral-oil-spread and regular agar plate			
Sampling time (min)	5	10	20
Indoor bacterial aerosol	0.171	0.007	0.009
Indoor fungal aerosols	0.243	0.003	0.003
Outdoor bacterial aerosols	0.002	0.000	0.000
Outdoor fungal aerosols	0.007	0.000	0.000
*B. subtilis* aerosols	0.002	0.001	0.000
*E. coli* aerosols	0.008	0.003	0.000

N = 3, p-values (independent sample t-test) When Andersen six-stage sampler is used with mineral-oil-spread and regular agar plate						
Andersen sampler Stages	1 st	2 nd	3 rd	4 th	5 th	6 th
Indoor bacterial aerosols	0.000	0.001	0.000	0.004	0.016	0.040
Indoor fungal aerosols	0.132	0.001	0.005	0.012	0.048	0.390
Outdoor bacterial aerosols	0.001	0.000	0.001	0.000	0.003	0.000
Outdoor fungal aerosols	0.573	0.253	0.931	0.397	0.097	0.369

The mineral oil film plated was about 180 µm thick, therefore the bioaerosol particles can be embedded into the layer, thus lowering the desiccation effects. Due to the non-evaporating attributes of mineral oil, the agar surface is also protected from the desiccation, accordingly reducing the particle bounce. In addition, the mineral oil layer is softer than the agar surface, thus reducing the relevant impaction stress. Despite the sampling desiccation stress, i.e., amount of air impacted on agar surface per unit area per unit time[23], remained similar for the stages of the Andersen sampler operated at 28.3 L/min, each stage has a different impaction velocity: 24 (6 th stage), 12.8 (5^th^ stage), 5.3 (4^th^ stage), 3.0 (3^rd^ stage), 1.8 (2^nd^ stage) and 1.1 (1^st^ stage) m/s, thus presenting different impaction stress. Larger bacterial particles tend to sustain higher impaction stress compared to smaller ones given similar impaction velocity. Accordingly, different stages of the Andersen sampler impart different impaction stress on the bioaerosol particles. Thus, use of mineral oil can have a greater protective effect for larger particles from the impaction stress than smaller ones. For different stages of the sampler, different size particles were collected because of their different cutoff sizes, thus the species collected onto different stages might be also different. Different species respond to the sampling stress differently. However, in natural environments bacterial particles could appear in aggregates or harbor on larger non-biological particles such that they could be collected onto upper stages of the sampler. This possibility could make differentiating the aggregates from their single cells difficult with respect to the recovery. Besides, different environments could also have different microbial species compositions. In addition, particles collected onto each stage of the Andersen six-stage sampler have stayed in the sampling line for different amount of time, and due to the particle bounce certain particles might have been impacted more than once too. Given all these influencing factors, the enhancement trend for different stages of the Andersen six-stage sampler by the mineral-oil-spread agar plate is rather difficult to predict.

**Figure 2:**
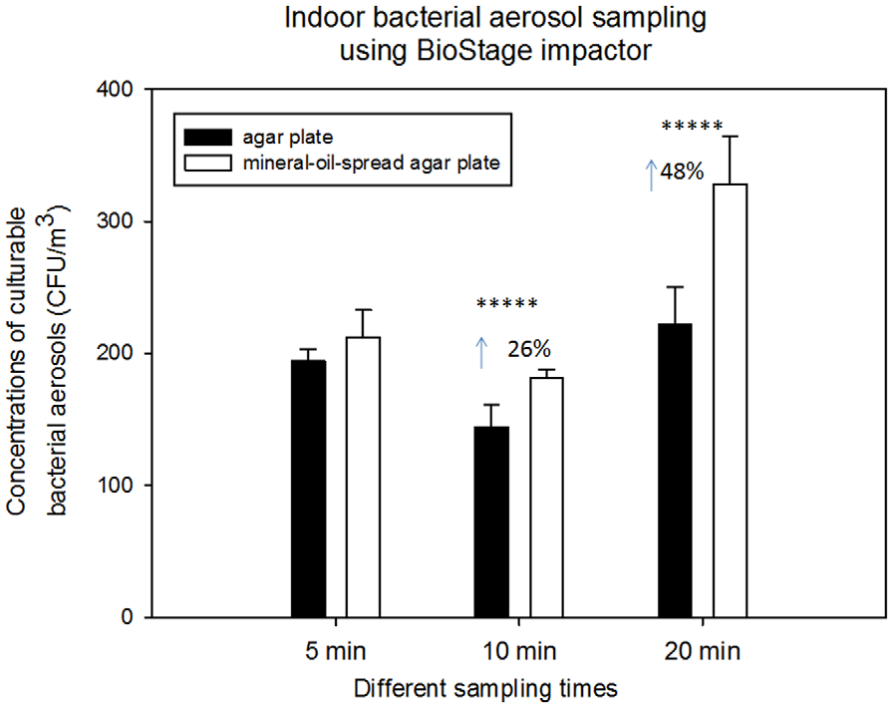
Biological collection efficiencies of BioStage impactor together with agar plate and mineral-oil-spread agar plate in sampling the total bacterial aerosols under different sampling times (5, 10 and 20 min) in an indoor environment; 100 µL mineral oil was evenly spread onto entire agar plate; 30 mL agar was used to fill the agar plate; data points represent averages and standard deviations of three independent sampling experiments;*****indicates a statistically significant difference.

**Figure 3:**
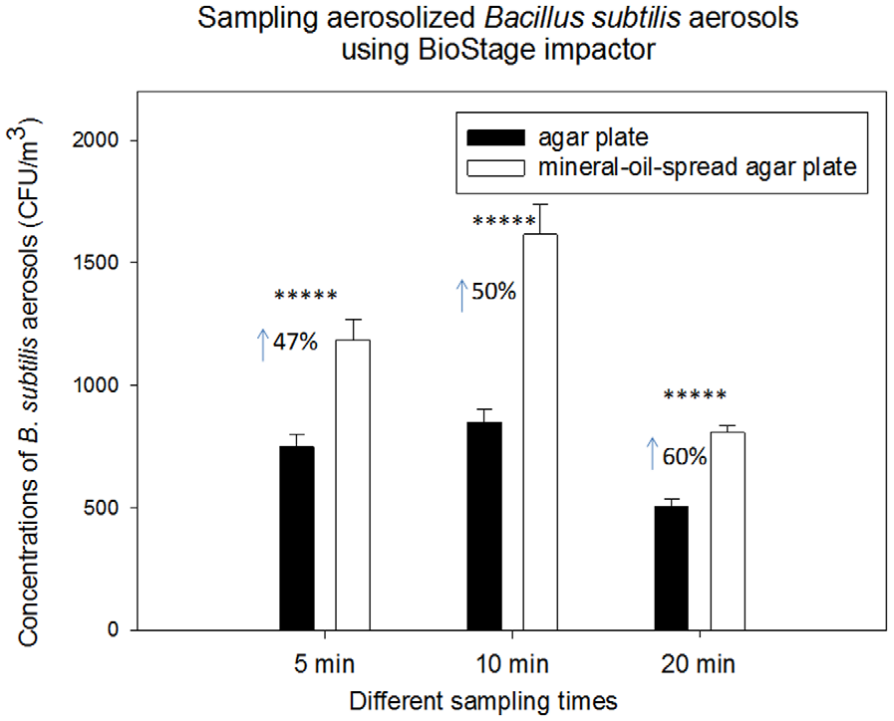
Biological collection efficiencies of BioStage impactor together with agar plate and mineral-oil-spread agar plate in sampling aerosolized *B. subtilis* aerosols under different sampling times (5, 10 and 20 min); 100 µL mineral oil was evenly spread onto entire agar plate; 30 mL agar was used to fill the agar plate; data points represent averages and standard deviations of three independent sampling experiments;***** indicates a statistically significant difference.

**Figure 4:**
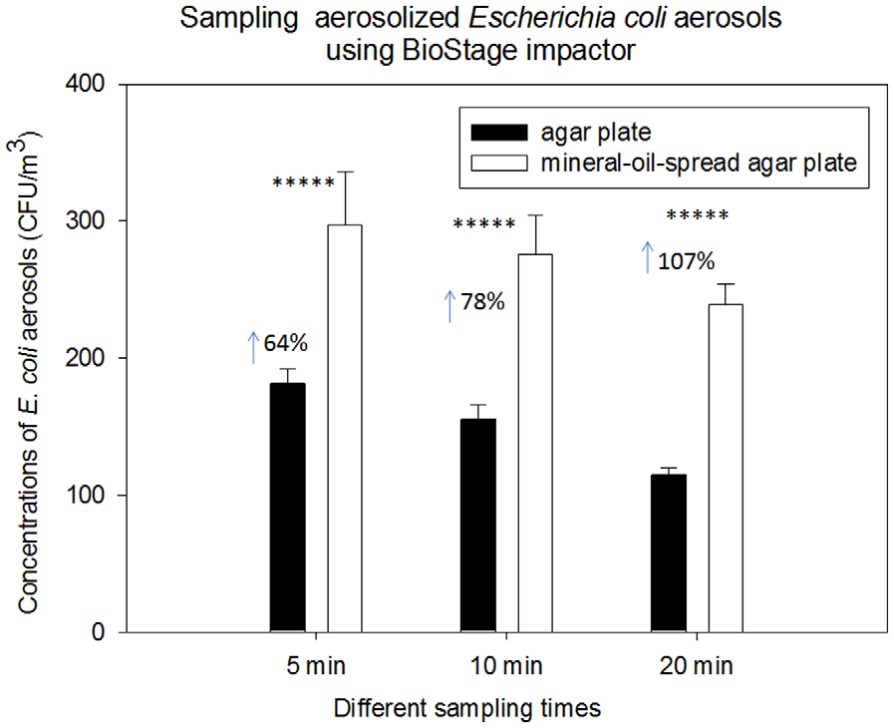
Biological collection efficiencies of BioStage impactor together with agar plate and mineral-oil-spread agar plate in sampling aerosolized *E. coli* aerosols under different sampling times (5, 10 and 20 min); 100 µL mineral oil was evenly spread onto entire agar plate; 30 mL agar was used to fill the agar plate; data points represent averages and standard deviations of three independent sampling experiments;***** indicates a statistically significant difference.

To investigate the effects of sampling time on the performances of mineral-oil-spread agar plates, we used the BioStage impactor to sample environmental bacterial aerosols, aerosolized *B. subtilis* and *E. coli* for 5, 10 and 20 min. The relevant results are shown in Figures 2, 3, and 4, S2 (Supporting Information). As observed in Figure 2, increasing sampling time resulted in increased enhancements from about 26% at 10 min to about 50% at 20 min (p-values = 0.007, 0.009) except for 5 min when mineral-oil-spread agar plates are used for sampling indoor bacterial aerosols. Unexpectedly, higher bacterial aerosol concentration was observed for 20 min as shown in Figure 2, which was likely due to the sudden environmental bioaerosol fluctuations. Similar results were obtained when the BioSatge impactor was used to collect outdoor bacterial aerosols under different sampling times (5, 10 and 20 min) as shown in Figure S2 (Supporting Information). Different from indoor sampling, all sampling times for outdoors were found to result in a statistical difference between mineral oil and agar plates (p-values<0.05 shown in Table 1). Compared to indoor bacterial aerosols sampling, more enhancements were observed for outdoor bacterial aerosol sampling, especially for 20 min sampling, about 2 times protection effects obtained for outdoor bacterial aerosols by use of mineral oil. This might be due to species compositions and their different sensitivity to the sampling stress in different environments. In different environments, Gram-positive (G+) and Gram-negative (G-) bacteria might account for different percentages, and they could respond to the sampling stress differently. Accordingly, different enhancements are expected by use of the mineral oil. In addition, the observed differences between indoors and outdoors might be also attributed to their different humidity levels: 46% for indoors and 36% for outdoors.

When sampling aerosolized *B. subtilis* and *E. coli* under different sampling times, similar results were observed as shown in Figures 3 and 4. Regardless of the sampling time, use of mineral-oil-spread agar plates was shown to substantially enhance the recovery rates of both species compared to the regular ones without the mineral oil plated (p-values<0.05 shown in Table 1). In general, for *B. subtilis* aerosols increasing sampling time was observed to lead to a decrease of aerosolized culturable bacterial aerosol counts collected by the regular agar plate due to the prolonged sampling desiccation; however for mineral-oil-spread agar plates increasing sampling time resulted in an increased recovery rate of bioaerosols, e.g., from 47% increase at 5 min to 60% at 20 min. This on the other hand indicates that the sampling stress causes a large number of bacteria cells to become non-culturable, while use of mineral oil can effectively minimize such side effects. Similar to *B. subtilis*, increasing sampling time resulted in increased recovery rates for *E. coli* aerosols, e.g., from 64% increase at 5 min to 107% at 20 min, when mineral-oil-spread agar plates used. In general, higher enhancement rates were observed for *E. coli* than *B. subtilis* at all sampling times by mineral-oil-spread agar plates. *E. coli* is a Gram-negative bacteria, more sensitive to environmental stress compared to Gram-positive *B. subtilis*, accordingly use of mineral oil had more protective effects on *E. coli*. In general, increasing sampling time also led to the decrease of culturable *E. coli* and *B. subtilis* counts given only mineral-oil-spread agar plates used. For environmental bioaerosols, the enhancement was found relatively lower (not statistically significant) for 5 min sampling time, which was possibly due to less particle bounce and desiccation. In contrast, for freshly aerosolized bacterial aerosols the enhancement was also found statistically significant more than 40% increase for the same sampling time. The difference might be due to the initial viability of the bioaerosols being sampled, i.e., high viability for freshly aerosolized bioaerosols and low viability for environmental ones.

Continuous sampling desiccation could also cause the agar to be desiccated, thus exposing those bioaerosols already collected, while mineral oil would not desiccate and evaporate[21]. The sampling desiccation for bioaerosols has been observed in many studies [18]-[20], [23], [24]. One study also indicated that the decreases in bioaerosol recovery by several portable microbial samplers were attributed to the desiccation of the agar plate (causing particle bounce) and those already collected bioaerosol particles [20]. Use of mineral oil can minimize the relevant desiccation problems due to its non-evaporating attributes. Previously, we have demonstrated that portable BioStage impactor reported higher culturable bacteria and fungal concentrations under 5 min sampling than the RCS High Flow due to the higher desiccation effects of the RCS High Flow sampler, about 8 times higher than the BioStage impactor [23]. In our recent study, we have shown that use of mineral-oil-filled agar strip resulted in about 4–12 times higher culturable bacterial aerosol concentration compared to the use of agar strips with RCS High Flow sampler[22]. Use of the mineral-oil-spread agar platewith the Andersen type sampler significantly reduced the sampling stress and particle bounce regardless of the species and environments.

**Figure 5:**
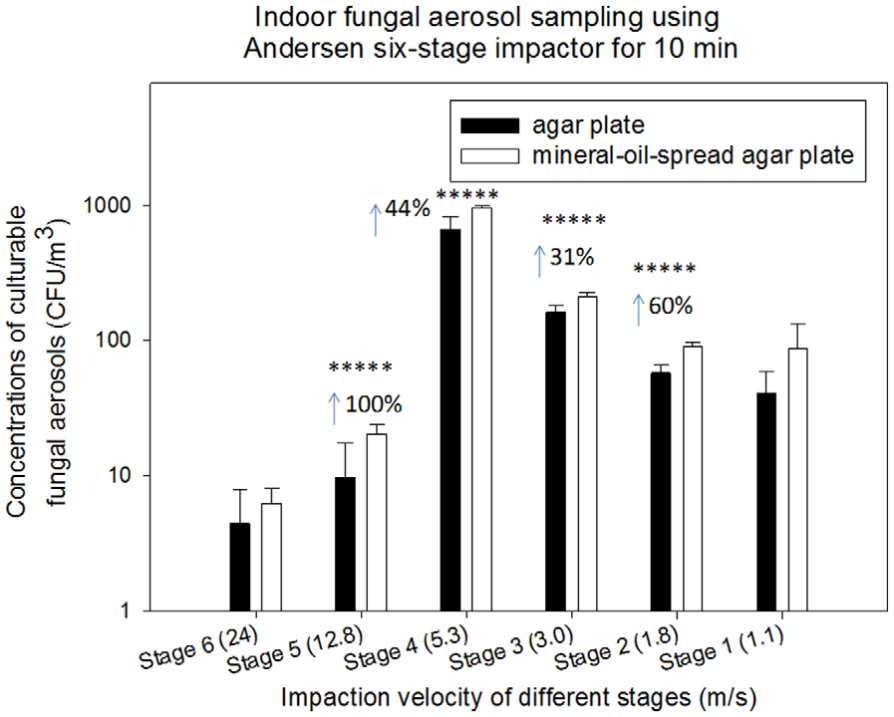
Size specific biological collection efficiencies of Andersen six-stage impactor together with agar plate and mineral-oil-spread agar plate in sampling fungal aerosols at a sampling time of 10 min in an indoor environment; 100 µL mineral oil was evenly spread onto entire agar plate; 30 mL agar was used to fill the agar plate; data points represent averages and standard deviations of three independent sampling experiments;***** indicates a statistically significant difference.

**Figure 6:**
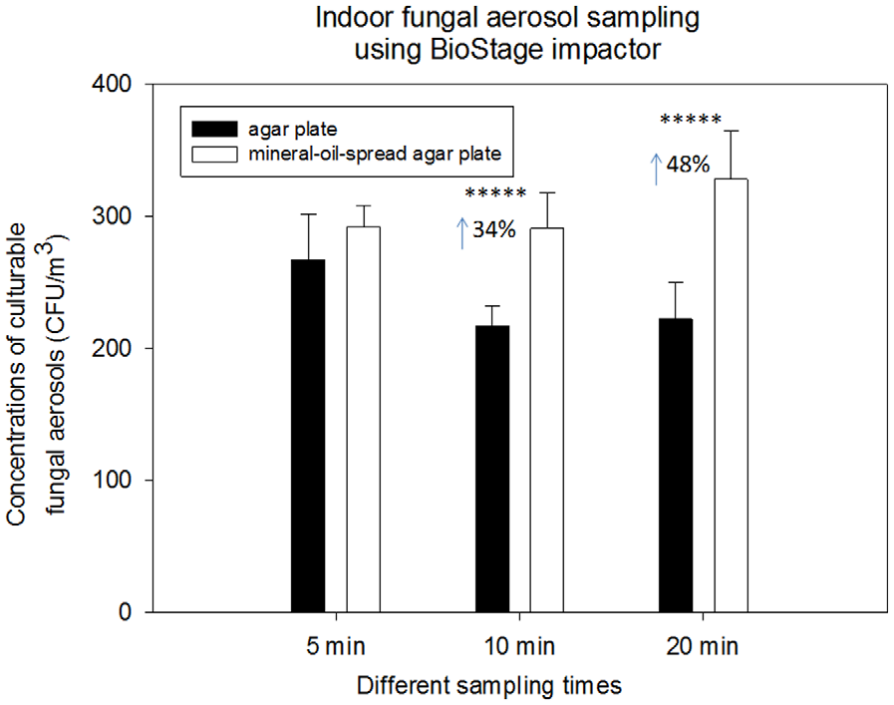
Biological collection efficiencies of BioStage impactor together with agar plate and mineral-oil-spread agar plate in sampling the total fungal aerosols under different sampling times (5, 10 and 20 min) in an indoor environment; 100 µL mineral oil was evenly spread onto entire agar plate; 30 mL agar was used to fill the agar plate; data points represent averages and standard deviations of three independent sampling experiments;***** indicates a statistically significant difference.

Besides, we also performed similar experiments with fungal aerosols using the Andersen six-stage and the BioStage impactor in the same environments, and the results are shown in Figures 5 and 6 and Figures S3 and S4 (Supporting Information). As observed in Figure 5 for indoor environment, different from the bacterial aerosol distribution shown in Figure 1, fungal aerosol distribution was found skewed around stage 3 and stage 4 (their cutoff size are 3.3 and 2.1 µm, respectively) for the Andersen six-stage sampler. In general, similar to bacterial aerosol sampling experiments, use of mineral-oil-spread agar plates was also observed to greatly enhance the fungal aerosol sampling by the Andersen six-stage sampler except for stage 1 (p-value = 0.13) and 6 (p-value = 0.39). And pronounced protective effects were found with stage 5 (100%, p-value = 0.048) and stage 2 (60%, p-value = 0.001). Similar to bacterial aerosols, the observed enhancements varied with stages, which were likely due to their different size distribution and species compositions. Unexpectedly, no enhancements were observed for outdoor fungal aerosol sampling with the Andersen six-stage sampler (p-values>0.05 shown in Table 1) as shown in Figure S3 (Supporting Information). With respect to the BioStage impactor for indoor fungal aerosol sampling, the difference was not statistically significant for 5 min (p-value = 0.243), while for 10 and 20 min, 34% and 48% enhancements were observed, respectively. Different from indoors, all sampling times for outdoor fungal aerosols with the BioStage impactor resulted in a statistical difference between mineral oil and agar plates (p-values<0.05 shown in Table 1) as observed in Figure S4 (Supporting Information). The difference between indoor and outdoor fungal aerosol samplings was likely due to their different particle size, species compositions and their sensitivity to the further stress.

However, the effects of sampling time on the fungal bioaerosol recovery were found not statistically significant both for regular agar(p-values =  0.87) and mineral oil (p-value =  0.879) plates with the BioStage impactor by independent sample t-tests. These results suggest that fungal aerosols might appear to be more desiccation resistant with less particle bounce, and use of mineral oil might not significantly improve their recoveries from pure desiccation effects given the same impaction velocity. Previously, it was also indicated that extended sampling time up to 6 h did not affect the culturability of certain fungal species by the filtration sampling method at a composting site [25]. In another study, fungal spores were also shown desiccation resistant as extended sampling did not reduce the culturability, instead increasing sampling time and flow rate resulted in decreased fungal spore recoveries for AGI-30 [26]. The observed low recovery for fungal spores with AGI-30 sampler in their study is likely due to the high impaction stress caused by the impingement process. In our previous study, we observed that electrostatic sampling that has several orders magnitude lower impaction velocity obtained richer fungal species than other samplers such as the BioStage impactor with higher impaction velocity[19]. These results suggest that fungal aerosols might be more susceptible to high impaction stress than desiccation.

Different from the Andersen six-stage sampler, the BioStage impactor collects all particles larger than 0.65 µm (its cutoff size), thus the enhancement observed was for all bacterial aerosols. Increasing sampling time would certainly increase the desiccation effects, and as discussed use of mineral oil could minimize such effects since the collected bacterial particles can be embedded into the mineral oil, thus adding a layer of protection. Compared to the last stage of the Andersen six-stage sampler for 10 min sampling, the enhancement rate for the BIoStage impactor appeared slightly smaller. This could be due to the differences in the bioaerosol particles being collected by the same stage of two different samplers given the impaction stress remained the same (the same impaction velocity 24 m/s). For the last stage of the Andersen sampler, bioaerosol particles being collected should be smaller ones (larger ones already collected by 1–5 stages), while those being collected by the BioStage impactor include all bioaerosol particles. In addition, the bioaerosol particles being collected by the last stage of the Andersen sampler have stayed in the sampling line for longer time compared to those for the BioStage impactor, thus sustaining more sampling injuries. Besides, bioaerosol particles being collected by the last stage of the Andersen sampler might have been impacted several times before their collection due to the particle bounce on the agar surface. As a result of these differences, the bioaerosol particles might have experienced more stress for last stage of the Andersen sampler compared to the BioStage impactor. While use of mineral oil can effectively minimize such effects, accordingly higher enhancement was observed for the last stage of the Andersen sampler than the BioStage impactor when comparing their samplings for 10 min.

**Figure 7:**
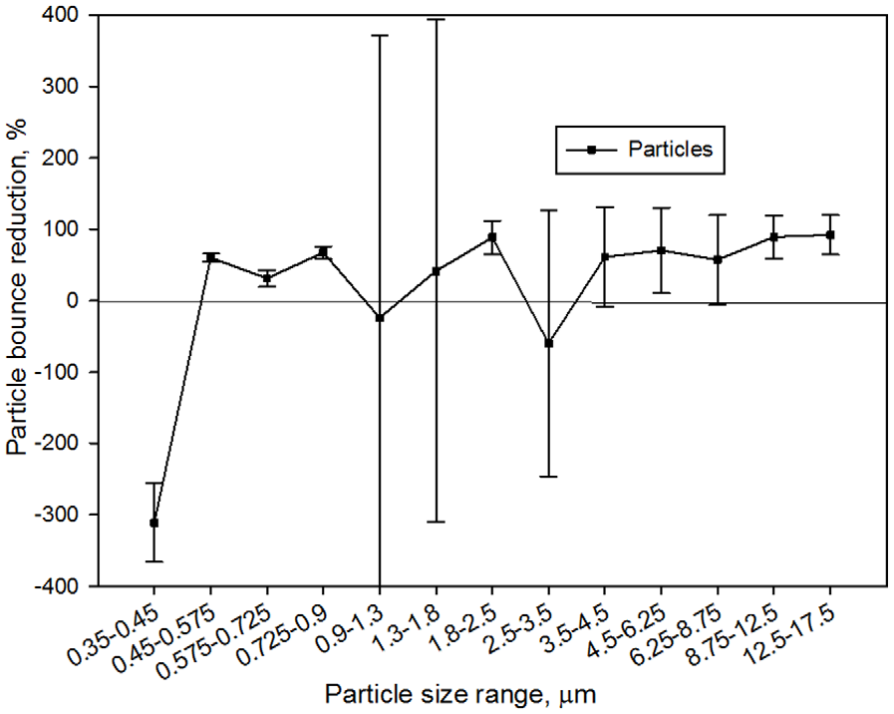
Indoor particle bounce reductions rates using mineral-oil-spread agar plate in replace of agar plate; the particle concentration levels were monitored using an optical particle counter; data points represent averages and standard deviations of 20 min continuous data; a negative value suggests an increase in particle bounce.

Our results with OPC monitoring suggest that use of mineral oil can result in significant reductions of particle bounce as observed in Figure 7. Excluding those negative values (for smaller particles, i.e., 0.35–0. 45 µm) and those with high variations due to sudden environmental fluctuations, the average particle reduction rate for all particles was calculated about 66% for a sampling time of 10 min. As discussed, for the Andersen sampler, due to the particle bounce many bioaerosol particles have been impacted more than once, thus sustaining more impaction injuries. However, for the BioStage impactor it is only one time impaction on the agar plate. Accordingly, more enhancements by mineral-oil-spread agar plates were observed with the last stage of the Andersen type sampler than the BioStage impactor. The observed enhancements for biaoerosol recovery using mineral-oil-spread agar plates here are primarily due to the reduced sampling stress (impaction and desiccation) and particle bounce problems.

Overall, experimental results in this work showed that use of mineral oil can sustain prolonged sampling time without reduced culturable bioaerosol recoveries when Andersen type samplers are used. As indicated by the results from the Andersen sampler and the BioStage impactor, the recovery enhancement was shown to depend on bioaerosol size, type, sampling time and environment. It appears that more fungal recoveries were attributed to the reduced impaction stress, while those for bacteria were likely arising from impaction, desiccation and particle bounce reduction by mineral-oil-spread agar plate. Future studies might need to be conducted with more pure bacterial species and different amount of mineral oil. Uniform oil plating on the agar plate might also further increase the bioaerosol recoveries for impaction based sampling technique. Besides, use of selective medium for impaction and desiccation injuries can identify the main contributing factors for improved bioaerosol recoveries using mineral-oil-spread agar plates under different conditions. Previously, high volume portable agar-plate based microbial samplers have been investigated for sampling both aerosolized and environmental bioaerosols[16], [17], and one recent study indicated that the sampling time for these portable samplers should be as short as possible to avoid the bioaerosol particle bounce and desiccation problems [20]. Importantly, the results from this work indicated that use of mineral-oil-spread agar plate can eliminate such restrictions. Therefore, the developed technique can extend the capability of high volume portable sampler to collect bioaerosols over extended time duration, especially for those in low concentration levels.

**Figure 8:**
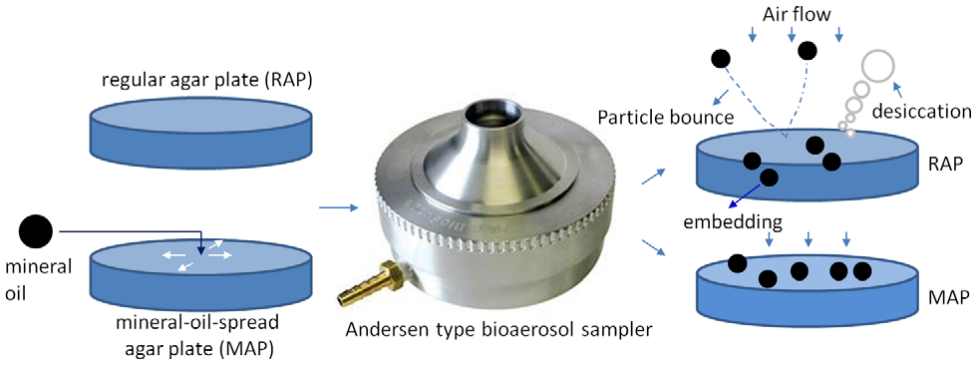
The illustration of use of mineral-oil-spread agar plate in improving the culturable bioaerosol sampling by the Andersen type sampler.

Andersen type impactor has been long used as a standard bioaerosol sampler since its invention in 1950s, and extensively applied in various applications. However, to our knowledge subsequent improvements for its problems such as particle bounce and desiccation has not been attempted. Here, we significantly improved its biological collection efficiencies by plating a layer of mineral oil (180 µm thick) on the regular plate as depicted in Figure 8. On the other hand, the results here also imply that traditional use of regular plate with the Andersen type impactors significantly underestimates actual bioaerosol loads. Application of mineral oil layer on the agar plate surface can substantially minimize the longstanding problems such as particle bounce and desiccation associated with agar-based bioaerosol sampling. In addition, use of mineral-oil-layer could also enable the increase of impaction velocity for increasing physical collection efficiency in future developments of impaction-based samplers. Here, only mineral oil was tested, and other type of non-evaporating collection media can be also spread on the agar plate for enhanced bioaerosol sampling. The developed technique here is very simple, and can be immediately applied to bioaerosol sampling with enhanced efficiency for various agar-based collection techniques.

## Supporting Information

Figure S1 Size specific biological collection efficiencies of Andersen six-stage impactor together with agar plate and mineral-oil-spread agar plate in sampling bacterial aerosols at a sampling time of 10 min in an outdoor environment; 100 µL mineral oil was evenly spread onto entire agar plate; 30 mL agar was used to fill the agar plate; data points represent averages and standard deviations of three independent sampling experiments; ***** indicates a statistically significant difference.
(JPG)

Figure S2 Biological collection efficiencies of BioStage impactor together with agar plate and mineral-oil-spread agar plate in sampling the total bacterial aerosols under different sampling times (5, 10 and 20 min) in an outdoor environment; 100 µL mineral oil was evenly spread onto entire agar plate; 30 mL agar was used to fill the agar plate; data points represent averages and standard deviations of three independent sampling experiments; ***** indicates a statistically significant difference.
(JPG)

Figure S3 **Size specific biological collection efficiencies of Andersen six-stage impactor together with agar plate and mineral-oil-spread agar plate in sampling fungal aerosols at a sampling time of 10 min in an outdoor environment; 100 µL mineral oil was evenly spread onto entire agar plate; 30 mL agar was used to fill the agar plate; data points represent averages and standard deviations of three independent sampling experiments.**
(JPG)

Figure S4 Biological collection efficiencies of BioStage impactor together with agar plate and mineral-oil-spread agar plate in sampling the total fungal aerosols under different sampling times (5, 10 and 20 min) in an outdoor environment; 100 µL mineral oil was evenly spread onto entire agar plate; 30 mL agar was used to fill the agar plate; data points represent averages and standard deviations of three independent sampling experiments; ***** indicates a statistically significant difference.
(JPG)
