# Supplementary figures and images for: Enhancing Bioaerosol Sampling by Andersen Impactors Using Mineral-Oil-Spread Agar Plate
Source: PLoS One. 2013 Feb 27;8(2):e56896. doi: 10.1371/journal.pone.0056896 (PMC3584084; doi:10.1371/journal.pone.0056896)

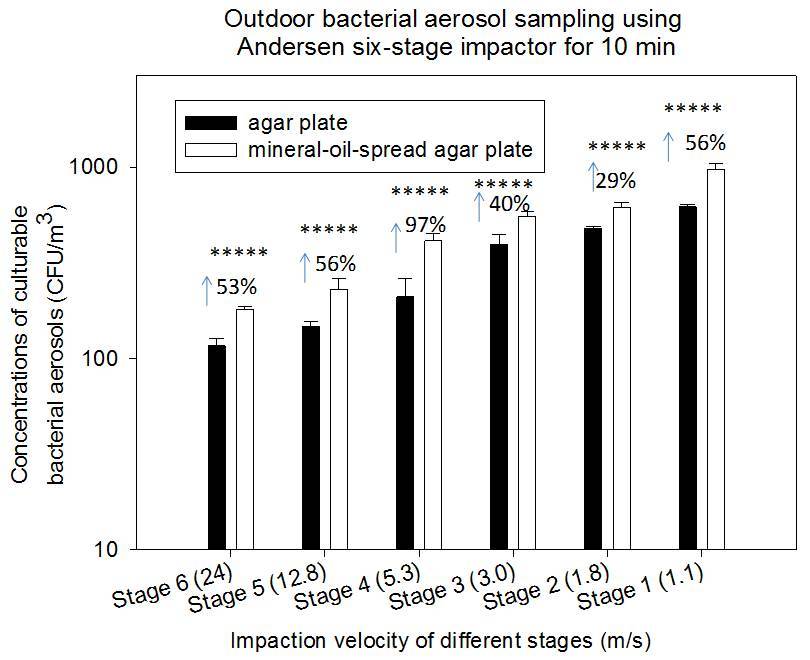

Supplement: Figure S1 — Size specific biological collection efficiencies of Andersen six-stage impactor together with agar plate and mineral-oil-spread agar plate in sampling bacterial aerosols at a sampling time of 10 min in an outdoor environment; 100 µL mineral oil was evenly spread onto entire agar plate; 30 mL agar was used to fill the agar plate; data points represent averages and standard deviations of three independent sampling experiments; ***** indicates a statistically significant difference. (JPG) [file pone.0056896.s001.jpg]

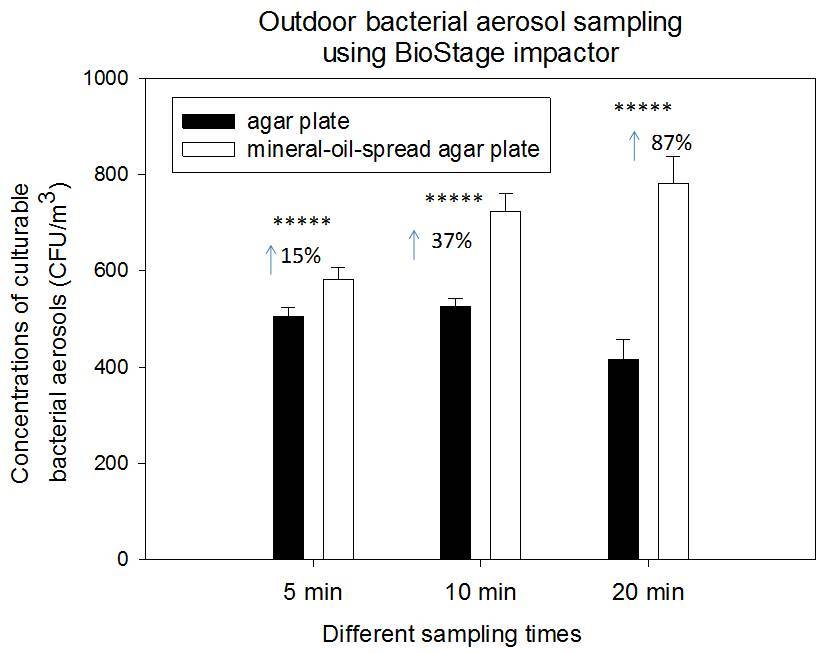

Supplement: Figure S2 — Biological collection efficiencies of BioStage impactor together with agar plate and mineral-oil-spread agar plate in sampling the total bacterial aerosols under different sampling times (5, 10 and 20 min) in an outdoor environment; 100 µL mineral oil was evenly spread onto entire agar plate; 30 mL agar was used to fill the agar plate; data points represent averages and standard deviations of three independent sampling experiments; ***** indicates a statistically significant difference. (JPG) [file pone.0056896.s002.jpg]

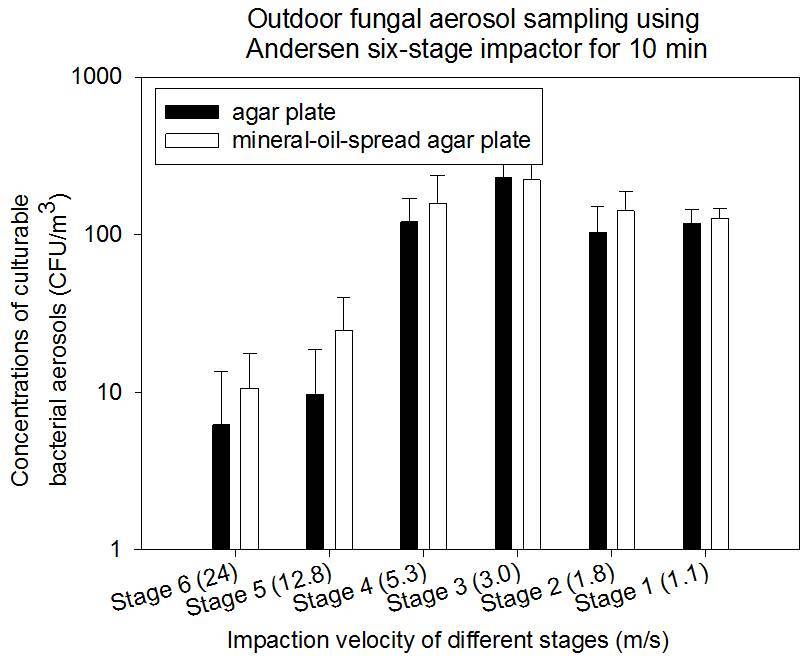

Supplement: Figure S3 — Size specific biological collection efficiencies of Andersen six-stage impactor together with agar plate and mineral-oil-spread agar plate in sampling fungal aerosols at a sampling time of 10 min in an outdoor environment; 100 µL mineral oil was evenly spread onto entire agar plate; 30 mL agar was used to fill the agar plate; data points represent averages and standard deviations of three independent sampling experiments. (JPG) [file pone.0056896.s003.jpg]

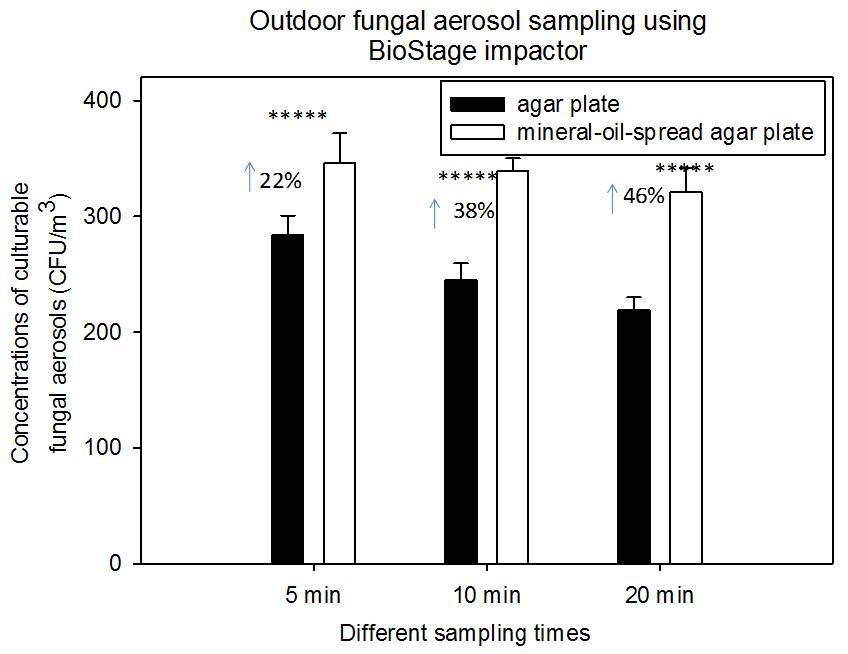

Supplement: Figure S4 — Biological collection efficiencies of BioStage impactor together with agar plate and mineral-oil-spread agar plate in sampling the total fungal aerosols under different sampling times (5, 10 and 20 min) in an outdoor environment; 100 µL mineral oil was evenly spread onto entire agar plate; 30 mL agar was used to fill the agar plate; data points represent averages and standard deviations of three independent sampling experiments; ***** indicates a statistically significant difference. (JPG) [file pone.0056896.s004.jpg]
